# Households’ willingness to pay and preferences for improved cook stoves in Ethiopia

**DOI:** 10.1007/s11356-021-14790-w

**Published:** 2021-06-12

**Authors:** Mekonnen Bersisa, Almas Heshmati, Alemu Mekonnen

**Affiliations:** 1 Department of Economics, Ambo University Woliso Campus, Woliso, Ethiopia; 2 Jönköping International Business School, Jönköping University, Room B5017, P.O. Box 1026, SE-551 11 Jönköping, Sweden; 3 Department of Economics, Addis Ababa University, Addis Ababa, Ethiopia

**Keywords:** Contingent valuation, Choice experiment, Cook stove, Energy-efficient technology, Ethiopia, C25, D12, Q51

## Abstract

This paper examines households’ preferences, willingness to pay, and determinants of adopting improved cook stoves in rural Ethiopia. The study uses primary household data selected randomly from three districts in Ethiopia’s Oromia region. The data was collected using a mix of contingent and choice experiment methods of valuation. The former used a double-bounded value elicitation method, while the latter used a fractional factorial design to efficiently generate an attribute and level combination for the improved cook stoves. The study also used various discrete choice models for data analysis and also used models which account for scale and preference heterogeneity. The findings show that the sample households were aware of the effects of using traditional cook stoves and the benefits of using improved cook stoves. However, they were constrained by the availability of the new technology and discouraged by the low-quality of the products that they had used so far. The estimated mean willingness to pay ranged from about 150 Birr to 350 Birr which is lower than the market price of the improved cook stoves. Emission reduction, reducing fire risks, and the durability of the cook stove positively affected its adoption, while price discouraged its use. Higher levels of education, higher incomes, non-farm employment, and having more livestock increased the probability of adopting the new gas stoves. The study recommends that policymakers and product designers should use the mean willingness to pay and marginal rate of substitution for the different attributes as a benchmark for product design and pricing that fit households’ preferences and ability to pay. The lower mean willingness to pay means that a public subsidizing policy is needed for effectively disseminating improved cook stoves in rural Ethiopia.

## Introduction

Energy is an important developmental tool at the forefront of the global economic and political agenda. Global environmental problems are largely related to energy use at different levels. Consequently, we observe growing efforts directed towards formulating and intervening in energy policies. Maintaining energy security, expanding access to renewable energy, disseminating energy-efficient technologies, and improving energy use efficiency are some of these policy interventions. However, the effectiveness of any policy intervention depends on societal readiness and support for the intervention (Ruiz-Mercado et al. 2011; Vigolo et al. 2018).

Expanding access to modern energy is tantamount to liberating 2.7 billion people globally who rely on inefficient and traditional energy sources such as firewood, charcoal, dung, and crop residuals for their energy needs. Access to affordable and reliable energy services in developing countries is fundamental for reducing poverty, improving health, increasing productivity, reducing environmental problems, and promoting economic growth. Modern energy and efficient energy use technologies have multiplier effects for development. They play substantial roles in the provision of clean water, sanitation, and healthcare and provision of reliable and efficient lighting, heating, cooking, and transport and telecommunication services. Despite this, in recent years, nearly 1.2 billion people (about 16% of the global population) in poor countries lacked access to electricity. Most of this electricity-deprived population lived in sub-Saharan Africa (SSA) and South Asia^1^ (Bhojvaid et al. 2014; Ruiz-Mercado et al. 2011; UNDP and WHO 2009; WEO 2016).

Evidence shows that the energy sector is one of the major contributors to greenhouse gas emissions. Indoor air pollution from biomass fuel use threatens health and claims lives of a substantial number of people in developing countries (WHO 2002). According to WEO (2016), each year around 3.5 million premature deaths are attributable to indoor air pollution. Ethiopia is also facing these problems. Traditional energy sources account for most of the residential energy use in the country. It has one of the lowest rates of diversified modern energy services. About 92% of the energy sources in the country come from biomass while oil accounts for about 7% and hydropower for about 1% of the energy sources. Moreover, the energy use pattern in the country shows that households account for 88% of the total energy consumption followed by industry (4%), transport (3%), and services and others (5%).^2^ Despite Ethiopia’s higher potential for the production of modern energy, only 25% of its population has access to electricity (Bersisa 2017; Dawit 2012; WEO 2013, 2016).

The problem is more serious for rural households in Ethiopia who rely more on biomass fuels. Lack of access to clean energy sources, health problems due to indoor air pollution, environmental degradation because of reliance on nature for collecting energy sources, and inefficiency of energy use technologies are well-known issues faced by rural households in Ethiopia. According to a WHO (2007) report, more than 50,000 deaths per year and 5% of the disease burden in the country were attributable to indoor air pollution.

In response to the challenges of development and the country’s aspirations of achieving sustainable development, the Government of Ethiopia has launched ambitious medium-term development plans, the latest of which is the Growth and Transformation Plan (GTP) launched in 2011. The country has a target of attaining lower middle-income status by 2025, and its growth path is aligned with the Climate-Resilient Green Economy (CRGE) strategy. Under this plan, the country has embarked on expanding its modern energy sources, and the energy sector is considered an important pillar for realizing green growth and accelerating development. Through its CRGE strategy, Ethiopia has shown the importance of addressing households’ energy demands including expanding renewable energy sources and promoting clean energy technologies (for example, dissemination of 31 million efficient cook stoves by 2030). Promoting efficient stoves is a part of fast-track initiatives for reducing greenhouse gas emissions and emissions from deforestation and forest degradation (FDRE 2011a, 2011b). Nevertheless, the effectiveness of any policy intervention largely depends on consumers’ preferences and willingness to pay for the improvements, their adaptation to a new environment, and continuous innovations in cook stoves and their dissemination for which policy interventions are needed (Ruiz-Mercado et al. 2011; Vigolo et al. 2018).

Vast literature evaluates households’ preferences and willingness to pay for energy-related interventions, but there are limited studies on these issues in developing countries. The few studies that are available focus on willingness to pay for electricity connections, improved cook stove adoption, and different types of fuel in Kenya, India, and Ethiopia (Abdullah and Jeanty 2011; Bhojvaid et al. 2014; Kooser 2014; Kroon et al. 2014; Takama et al. 2011). Studies in Ethiopia are limited to urban areas, and they are methodologically unable to account for preferences and scale heterogeneity in estimating willingness to pay for improved cook stoves. The problem is less studied in rural areas where a majority of the households in developing countries reside with very different livelihood setups. Besides, people make purchase decisions for a commodity on the basis of some of its features. In this case, the decision-makers claim “attribute non-attendance.” Attribute non-attendance is when respondents ignore a given attribute and its associated level while evaluating an alternative package of attributes. These are behavioral responses and ignoring them in an analysis will bias the estimated mean willingness to pay (Campbell et al. 2011; Hensher and Greene 2010).

The main contributions of this study to existing literature include extending the study to rural areas, incorporating more attributes of improved cook stoves, using a combination of a choice experiment and a contingent valuation method, examining attribute non-attendance, testing different tools for reducing hypothetical bias, and employing discrete choice models which account for preference heterogeneity and scale heterogeneity. In doing so, the study addresses the following research questions: (i) to what extent are rural households aware of the negative effects of using traditional cook stoves and their preference for improved cook stoves?, (ii) what are the cook stove-specific attributes and the socioeconomic determinants of adopting these improved stoves?, (iii) can a household afford to buy an improved cook stove?, (iv) do households exhibit preference heterogeneity in choosing the improved cook stove?, and (v) does a household show attribute non-attendance in choosing an improved cook stove?

To address these questions, the study focuses on examining the preferences, willingness to pay, and determinants of the use of improved cook stoves among rural households in Ethiopia thus providing relevant information for rural energy planning and policy.

The rest of this paper is organized as follows. The “Literature review” section presents a review of existing studies on the theory of non-market valuation techniques, cost-benefit analyses, the non-market valuation method, and a theoretical framework of the choice models. It shows the gap in literature on the valuation of improved cook stoves and willingness to pay estimations. The “Data and estimation methodology” section gives details of the data and estimation methodology used. The “Results and discussion” section discusses the results of the study, while the final section gives a conclusion and policy recommendations.

## Literature review

In reviewing the literature, the focus is on the theory of non-market valuation and cost-benefit analysis, theoretical framework and valuation techniques, and empirical literature on issues related to energy use, adoption of energy-efficient technologies, and households’ preferences and willingness to pay for energy-efficient technologies.

Non-market valuation methods have become an important tool for valuing non-marketed resources (Haab and McConnell 2002; Hanemann et al. 1991). These methods have also been extended to the valuation of marketed goods and services where the conventional market fails to reflect their true values. When there is market failure, market price provides a wrong signal about the economic value of a good or service. The market fails due to the existence of the perverse effect of production and consumption, information asymmetry, lack of well-defined property rights, and existence of public goods (Haab and McConnell 2002; Perman et al. 2003). On the other hand, no market exists for some goods and services making it difficult, if not impossible, to estimate the economic value of such goods. Often, many analytical approaches of project evaluation require some considerations for estimating values in terms of costs and benefits. A peculiar aspect of the cost-benefit analysis is that it requires that the advantages and disadvantages of a project be reduced to numbers which complicates the valuation. Costs of any project are easier to estimate. A more daunting task is estimating economic benefits. Economists’ non-market valuation techniques are a strong tool for addressing this problem (Ackerman and Heinzerling 2002; Haab and McConnell 2002; Perman et al. 2003).

Non-market valuation methods are generally categorized under two methods: stated preference and revealed preference methods. In stated preference methods, we ask people what they would like to pay or accept for a particular change to happen or not to happen. It is a direct method where people are asked to state their willingness to pay or their choice for a given proposed change. Stated preference methods can be used for preference/choice evaluation, demand analysis, and forecasting. The most frequently used methods here are contingent valuation, choice experiment, contingent ranking, and contingent rating (Haab and McConnell 2002).

Revealed preference methods are indirect non-market valuation methods. These are used for deriving economic value for non-market goods/services indirectly from individual decisions. They encompass travel costs, hedonic pricing, hedonic wages, and averting behavior methods. The contingent valuation method asks a sample of individuals about their willingness to pay for a hypothetically designed product (Carson 2012; Hanemann 1994). A choice experiment requires a sample of the respondents to make a series of choices from experimentally designed choice sets from which trade-offs between attributes and the marginal rate of substitution are estimated. Applications of choice experiment include economics of the environment, health, transport, marketing, and energy sectors (Adamowicz et al. 1998; Aizak and Nishimura 2008; Haab and McConnell 2002; Hensher et al. 2005).

Non-market valuation techniques are methods of attaching total economic value to goods or services. A non-market valuation exercise is grounded in the theory of welfare economics (Haab and McConnell 2002). Respondents evaluate the effects of any intervention, whether it is an improvement or deterioration on their welfare. An analytical tool that can be used for this is computing the welfare changes of such an intervention. One of the commonly used stated preference valuation techniques is choice experiment. A choice experiment involves following a choice modeling approach which is grounded in the traditional microeconomic theory of choice and decision theory (Hanley et al. 2001; McFadden 1986). The choice experiment approach combines the characteristic theory of value and the random utility theory (Lancaster 1966; McFadden 1974). The characteristic theory of value states that a consumer’s utility for a good is decomposable into the utility of the characteristics or attributes of the good (Hanley et al. 2001). Moreover, the utility derived from each alternative is assumed to be determined by preferences over the level of attributes provided by that alternative. The assumption that individuals derive utility from the characteristics of a good rather than from the good itself implies that a change in one of the characteristics (such as the price) may result in a discrete change from one good to another which will affect the probability of choosing a specific commodity on the margin (Hanley et al. 1998; Lancaster 1966).

In a choice experiment, a respondent is asked to choose the most preferred among a set of alternatives. Hence, the random utility theory is appropriate for modeling the choices as a function of attributes and attribute levels. In the random utility theory, an individual is assumed to make choices based on the attributes of the alternatives with some degree of randomness. The random utility theory says that the utility derived by individuals from their choice is not directly observable, but an indirect determination of preferences is possible and it decomposes the utility (U) function into a deterministic (V) and a stochastic part (ɛ). The stochastic part is assumed to follow a predetermined distribution (Brown and Walker 1989; Hanley et al. 2001; McFadden 1974).

There is vast empirical literature on issues related to energy use, adoption of energy-efficient technologies, and households’ preferences and willingness to pay for energy-efficient technologies. Since energy plays a crucial role in economic development and improving social welfare, it has been an area of active inquiry. Most of the studies available so far focus on the effectiveness of interventions, factors effecting energy use technology adoption, and the nexus between energy, poverty, and the environment (Akpalu et al. 2011; Alem et al. 2014; Amigun et al. 2011; Bersisa 2017). Existing literature also shows that individuals’ preferences and tests matter for the effectiveness of policy in promoting energy-efficient technologies. A considerable amount of available studies use stated preference methods for estimating the value of the improvements.

Using the contingent valuation methodology, Abdullah and Jeanty (2011) analyzed rural households’ willingness to pay (WTP) for electricity connections in Kenya. Their study used a double-bounded elicitation format to generate data from the sampled households. They used two econometric methods (parametric and non-parametric) for estimating WTP for an electricity connection and found that the mean WTP of the non-parametric estimation was lower than the mean WTP in the parametric approach. Furthermore, their study showed that respondents’ income, education level, interest in business, age, family size, and house ownership significantly affected WTP for an electricity connection. The study concluded that rural electrification programs should follow a bottom-up approach accounting for consumer preferences and the need for policy formulation.

Similarly, Takama et al. (2011) analyzed households’ willingness to pay and preferences for improved cook stoves in Ethiopia, Tanzania, and Mozambique. Their study emphasized the role of product-specific attributes (indoor smoke, safety, usage cost, and price of the stove) along with socioeconomic factors which are commonly used in literature as determinants in studies on improved cook stoves. Takama et al.’s study used the stated preference survey to examine household-level preferences for cooking fuels and stoves. Geographically, the study focused on the capital cities of the three countries to draw sample households: 200 from Addis Ababa, 564 from Dar es Salaam, and 402 from Maputa. Data generated using the stated preference survey was analyzed using a discrete choice analysis for examining the trade-off between the attributes of improved cook stoves. The study concluded that product-specific attributes were as important as households’ socioeconomic characteristics and should be given due emphasis in program design, developing products, and policymaking.

A comprehensive study by Bhojvaid et al. (2014) in rural India showed that households mean willingness to pay for improved cook stoves varied significantly according to stove-related attributes. Kroon et al. (2014) studied households’ preferences for fuels and willingness to pay for alternative cook stove technologies in Kenya. Kooser (2014) examined Ethiopian households’ preferences and willingness to pay for improved cook stoves. This study showed that adoption of improved cook stoves was affected by various cook stove–specific attributes and socioeconomic characteristics.

Jagger’s and Jumbe’s (2016) study in rural Malawi showed households’ preferences and willingness to adopt a locally produced improved cook stove. Their study used the discrete choice experiment on 383 households randomly selected in rural Malawi for getting their preferences for the locally produced improved cook stove or a package of sugar and salt with equivalent value. Their study showed that the availability of large crop residuals, long time devoted to the collection of fuel wood, awareness about the environmental impact of wood fuel, and peer-effect at the village level increased the odds of choosing the improved cook stove, while availability of a large labor force for fuel wood collection and experience with non-traditional cooking facilities decreased the odds of choosing the improved cook stove.

Using data from rural Guatemala, Bielecki and Wingenbach (2014) showed the importance of cultural and social perceptions in adopting improved cook stoves. Beyond the “triple benefits” of the improved cook stove — health benefits, preserving the local ecosystem, and greenhouse gas reduction — the study argued that the adoption of improved cook stoves was also influenced by other benefits of the stove such as lighting, heating, and becoming a social gathering.

Alem et al. (2014) used panel data for examining the adoption and dis-adoption of improved electric cook stoves in urban Ethiopia. Their study used three rounds of the Ethiopian Urban Socioeconomic Survey for examining the determinants of electric cook stove adoption and dis-adoption which shed light on the state of energy transition in the country. They found that the price electricity, price of fire wood, and access to credit significantly affected the adoption of electric stoves. However, the study did not include product-specific attributes as determinants of cook stove choice.

Benka-Coker et al. (2018) used different methods for examining the effectiveness, uptake, and scale-up potential of the ethanol Clean Cook stove in a refugee camp and urban settings in Ethiopia. Using the mixed secondary and qualitative primary data, their study applied the Reach, Effectiveness, Adoption, Implementation, and Maintenance (RE-AIM) framework for evaluating the effectiveness and sustainability of the ethanol cook stove intervention and the effectiveness of subsidized distribution of the stove for low-income groups. Their results showed that the improved cook stove had numerous benefits. The study concluded that there were complexities in promoting a new fuel for household cooking, existence of many obstacles, and stagnation in implementation. The study also showed that there was potential for scale-up and commercialization of the ethanol Clean Cook stove in Addis Ababa. It also showed the necessity of stabilizing ethanol supply, providing city-wide distribution infrastructure, and an affordably priced stove and fuel.

Similarly, Mamuye et al. (2018) did a study examining the emissions and fuel use performance and determinants of adopting improved cook stoves in Dodola, southeastern Ethiopia. Their study showed that the improved cook stove reduced CO, CO_2_, fine particulate matter, and time used for cooking compared to the traditional stove. The study also showed that household head’s sex, age, education level, and income were the determinants for the adoption of the improved cook stove. However, the study did not consider stove-specific attributes that could influence the adoption of the cook stove.

Vigolo et al. (2018) did a systematic literature review for identifying the drivers and barriers to improved cook stoves from a consumer behavior perspective. Their review identified seven categories of determinants of adoption of an improved cook stove: awareness of the risks associated with traditional cook stoves and the benefits of the improved cook stove; attitude towards the technology; economic factors; fuel availability; location; socio-demographics; and social and cultural influences. The study concluded that for the effectiveness of the improved cook stove technology adoption, policymakers and managers should pay due attention to the local context and its social and cultural dynamics.

A handful of studies have applied the stated preference methodology for estimating households’ willingness to pay for improved cook stoves. However, these studies have arrived at conflicting results. For instance, a study by Jeuland et al. (2015) using data from rural households in North India shows that households had strong baseline preferences for the traditional cook stove which will be an inhibiting factor for a wider adoption of the improved cook stove. The study recommends the need for a reinvigorated supply chain with complementary infrastructural investments, appropriate incentives for consumers, and continued applied research and knowledge generation for scaling up the distribution of the improved cook stove.

In sum, existing literature on the adoption of improved cook stoves shows the importance of stove-specific attributes. It also shows that a mix of these attributes greatly affects the adoption of improved cook stoves in addition to the socioeconomic characteristics of the adopters. However, the problem is less studied in rural areas. Moreover, to the best of our knowledge, non-attendance of one or more attributes in making a choice for an improved cook stove has not been studied in Ethiopia. Attribute non-attendance, where respondents ignore a given attribute, and its associated level while evaluating alternative packages of attributes are behavioral responses and hence ignoring them in an analysis will bias the estimated mean willingness to pay (Campbell et al. 2011; Hensher and Greene 2010; Scarpa et al. 2009). This paper contributes to existing literature by extending this study to rural areas, incorporating more attributes of the improved cook stove which fit in rural housing realities and testing the existence of attribute non-attendance. It methodologically uses a mix of different models to account for preferences and scale heterogeneity in choice decisions.

## Data and estimation methodology

This section first describes the data and then the estimation methodology. Primary data was generated using a survey of households by employing random sampling technique and the stated preference methodology. The study also used the choice experiment technique for a hypothetically designed improved cook stove scenario. The estimation methodology includes use of different discrete choice models for estimating WTP and its distribution. The marginal effects of attributes and mean WTP for improved cook stoves using multinomial logit model are computed. Determinants of WTP from contingent valuation method are identified and their effects on WTP estimated.

### Description of the survey techniques and experimental design

This study was conducted in three selected zones of the Oromia national regional state which is one of the nine regional states in Ethiopia. The selected zones are in the center of the country and in the central part of Oromia region. The selection of these three zones was based on maintaining the heterogeneity of the respondents. These selection criteria were used to account for respondents with different levels of access to forest resources which are important for biomass fuel use. The selected zones are North Showa, South West Showa, and the Finfinne Surrounding Oromia Special Zone. One district was randomly selected from each selected zone. The selected districts are Wachale, Bacho, and Sebetahawas. Eight kebeles^3^ were selected from these districts using cluster sampling to include people with different socioeconomic, demographics, and agro-climatic conditions in the sample.

Primary data was generated using a survey^4^ of 307 households by employing a multi-stage random sampling technique. Random sampling and sampling proportion to size were used for selecting the households from the selected kebeles. To collect information from selected households, face-to-face interviews were conducted with selected respondents. However, before doing the final survey, a pilot study was done for evaluating the appropriateness and clarity of the instruments and for determining the initial bid (price) used in the final survey. Feedback from the pilot study was incorporated in the final instrument. Five well-trained and experienced enumerators, closely monitored by two supervisors, were involved in the data collection process. The anonymized data was encoded using the SPSS software, and it was then transferred to different versions which are compatible with Stata and Nlogit used for the data analysis in this paper.

The data was collected using the stated preference methodology. It used two stated preference techniques: contingent valuation and choice experiment. For contingent valuation, a scenario in a carefully structured hypothetical market for an energy-efficient cook stove was designed, and households’ willingness to pay for such a product was generated using the double-bounded value elicitation format. This elicitation format was preferred as it fits marketing realities in developing countries where bargaining is common. A respondent was provided with a randomly selected initial bid (proposed price of an improved cook stove) with three bid levels of 75, 150, and 250 birr which was determined from the pilot survey and expert consultations. Depending on the response to the initial bid, a yes or no, the next bid was increased (decreased) by 50%. Following the final response (yes or no), the respondents were asked to state their maximum willingness to pay for the proposed improved cook stove.

The study also used the choice experiment technique for a hypothetically designed improved cook stove scenario. The choice experiment was used for identifying households’ preference for the different attributes of energy-efficient stoves. The respondents were asked to make a series of improved cook stove choices with different combinations of attributes which were mutually exclusive. Detailed information on both attribute-specific and socioeconomic characteristics was generated. In designing a choice experiment, one should consider various factors which can affect the choice of any specific cook stove. These factors can be categorized as stove-specific characteristics which include durability, ease of use, heat energy delivered, start-up time, the price of the stove, and the convenience of use. The second category is related to the risks associated with the use of the technology such as risk of explosion and increasing air pollution levels. The final category includes reliability of use due to different constraints like sustainable supply or availability of energy. Different types of energy-efficient cook stoves exist in Ethiopia albeit with low penetration rates. Mirt, Tikikil, Lakechi, Gonze, and the traditional three-stone cook stoves were considered for the design of the experiment. Of the cook stoves available in the country, the study primarily focused on the cook stove used for baking Enjera since it accounts for 50 to 60% of a household’s energy use (Bizzarri 2010).

Stated preference exercises involve a series of steps. One vital step in a choice experiment survey is determining the number of alternatives, attributes, attribute levels, and values to be considered. Traditionally, a stated preference survey relies on a binary choice for reducing the burden of choice making. Even though it is simple to handle, the binary choice approach has less policy relevance and pragmatism. Thus, extending the number of alternatives and attribute levels improves response quality; it increases the realism of the responses and has room for masking the aim of the study to avoid strategic bias (Hanley et al. 2001).

**Fig. 1:**
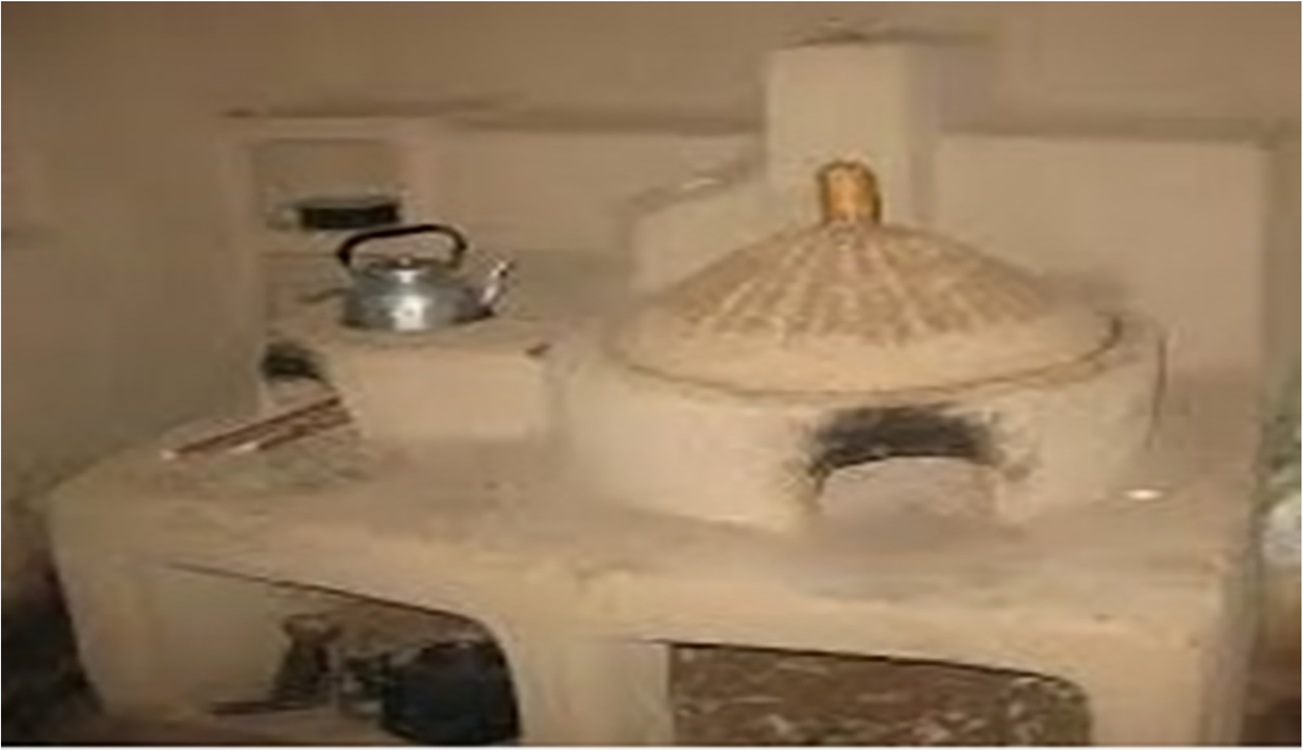
The proposed multipurpose improved cook stove

Hence, for estimating households’ willingness to pay and preferences for the improved cook stove in rural Ethiopia, different attributes of the improved cook stove were selected based on a literature survey, focus group discussions, pilot study, and expert consultations. Various attributes of the improved cook stove were identified. However, due to the complexity of the experimental design and households’ limited cognitive abilities in making choices, we limited the attributes included in the experiment to reducing indoor smoke/emissions, reducing risks of a fire, saving fuel, and durability and price of the improved stove. The proposed improvements to the cook stove were done in such a way that it became a multipurpose stove and could be used for baking Enjera, cooking wat, and boiling coffee. So far, this model has not been developed and distributed in Ethiopia. But making the stove multipurpose was meant to increase energy use efficiency and increase economies of scope and scale advantages in fuel wood use (see Fig. 1).

Along with selecting the attributes of the improved cook stove, it was also necessary to determine different levels corresponding to each attribute. Three levels were selected for each attribute of the improved cook stove. The starting points for level selection for each attribute were driven by empirical studies on the topic (Beyene et al. 2015). The attributes are as follows:

1. *Indoor smoke or emission reduction*: indoor smoke is an acute health problem that rural households face because they rely on traditional cook stoves and traditional energy sources. High indoor emission levels expose household members to different health risks. Thus, this program is designed to reduce the problems of indoor smoke because of using the traditional three-stone cook stove. The program integrates different packages with the improved cook stove to reduce emission levels by 40, 65, and 90%.
2. *Risk of use*: this program was designed in response to the different hazards of using the traditional cook stove. It involves designing a cook stove which reduces risks of a fire, burning, and explosions. It makes households safer and reduces related costs for prevention and curatives. Risk level is defined as low, medium, and high.
3. *Fuel saving*: shortage of fuel wood and higher costs of fuel and its environmental effects through deforestation are some of the problems related to fire wood collection and use. This program is meant to improve the stove’s efficiency and reducing fuel wood usage by 25, 45, and 65%.
4. *Durability of the stove:* frequent breakages of the stove expose a household to unnecessary costs. This program aims at producing a stove which can serve a household for different durations — 5, 15, or 20 years — depending on the material used in its production.
5. *Price of the stove:* it is unquestionable that price affects the demand for a product. Three price levels of 75, 150, and 250 birr were used in the survey to examine the effect of price on the demand for the stove and for the computation of marginal willingness to pay for each attribute (Table 1).

**Table 1:** Attributes and levels of the proposed improved cook stove

Attributes/stove type	Levels	Traditional stove
Indoor smoke/emission reduction	40, 65, and 90%	Status quo
Risk of use	Low, moderate, high	Status quo
Fuel wood saving	25, 45, and 65%	Status quo
Durability of the stove (in years)	5, 15, 20	Status quo
Price of the improved stove (in birr^a^)	75, 150, 250	Status quo

Source: Developed by the authors

^a^US$1 = 35.06 Ethiopian birr (ETB) on 7 August 2020.

Five attributes with three levels each were used for constructing the choice sets. The algorithm of experimental design of the R software in the discrete choice experiment was used for the experimental design and for testing the efficiency of the design. The SAS software was used for supplementing the experimental design and for testing the efficiency of the choice experiment’s design. With a D-efficiency of 97.8%, the orthogonal fractional factorial design was developed for the first alternative, reducing the original full factorial design of 243 (3^5^) possible combinations to 18. It was not feasible to present the full factorial design with all the possible treatment combinations as choice tasks. As a result, only a fraction of the total number of treatment combinations was selected. Optimum treatment combinations were selected such that all the attributes were statistically independent of each other (orthogonal). Finally, the 18 treatment combinations selected were divided into three versions to minimize inefficient selection in multiple choice tasks. The discrete choice experiment provided a panel of six choice sets for a given version (see Table 2). Each choice set consisted of two experimentally designed alternatives — labeled “option 1” and “option 2” — and a status quo alternative — labeled “no action” —which portrayed all the attributes of the currently in-use three-stone cook stove with no additional payment.

**Table 2:**
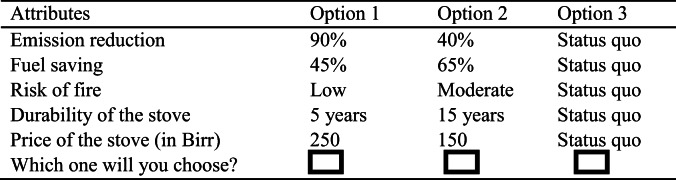
Sample choice sets used for the survey. Source: Designed by the authors.

**Fig. 2:**
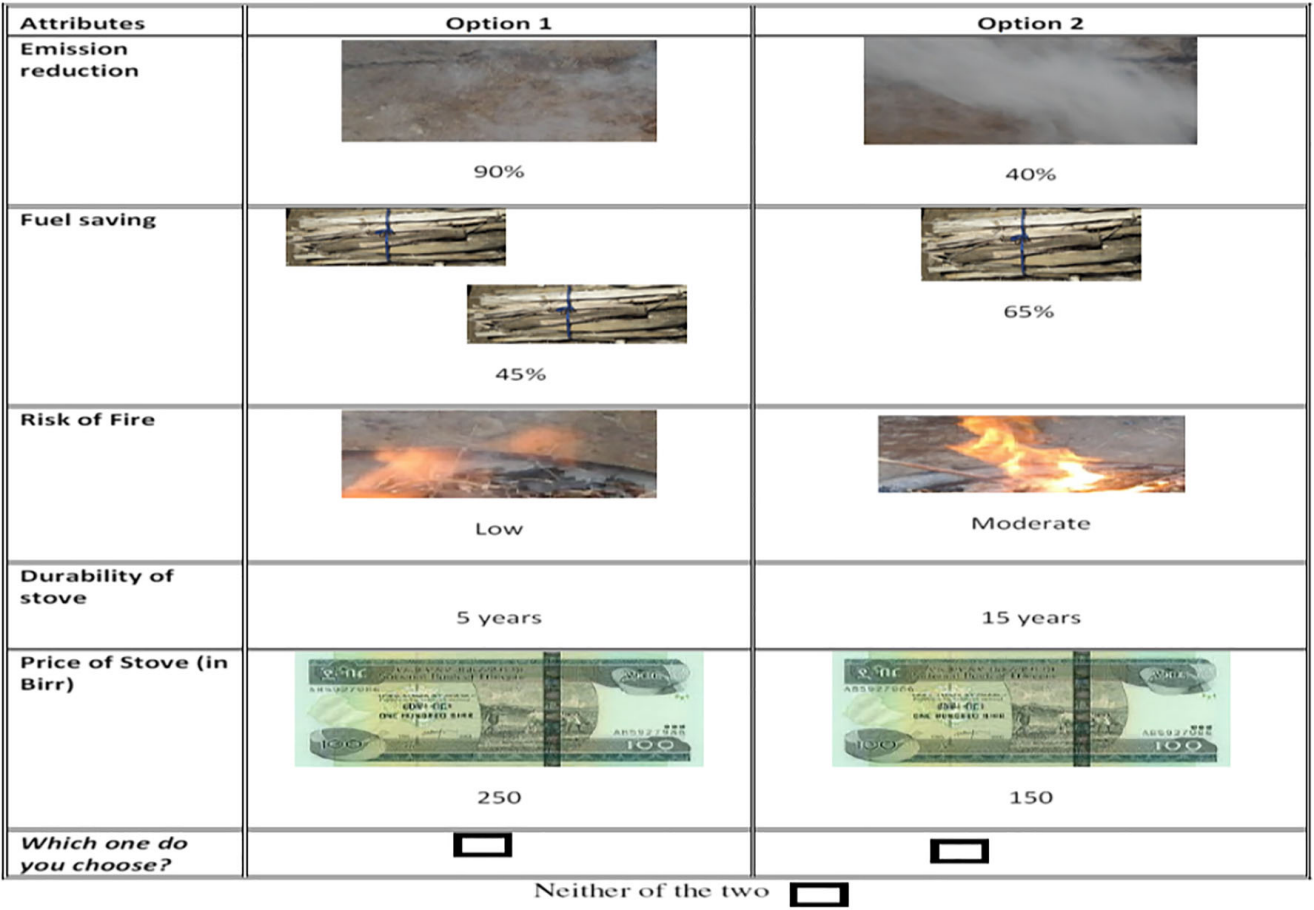
Sample choice card. Source: Designed by the authors

As we can see in Table 2, understanding improvements in each attribute in the choice sets could be difficult for some respondents. As the survey was conducted in rural areas where a majority of the respondents have low educational levels, it was necessary to supplement the choice sets by a choice card which diagrammatically represented the choice sets. To assist respondents and to make the choice task as simple as possible, choice cards were prepared for each choice set and presented to the respondents. A sample choice card is given in Fig. 2.

### Model specification and estimation methods

In this section, we introduce discrete choice models of multinomial logit, mixed multinomial logit, and scale heterogeneity multinomial logit that are used for analyzing choice experiment data. The analysis of the improved cook stove choices is further extended to the generalized mixed logit model. Models for estimating WTP distribution using analysis of the double-bounded response data from the contingent valuation method survey are introduced which uses interval data. The optimal class size is selected, and marginal WTP for improved cook stove’s attributes using multinomial logit model is computed. Determinants of WTP are identified and their effects on WTP estimated. Finally, mean WTP using different models is estimated. The multinomial model and its generalization are described below.

#### Models used for analyzing choice experiment data

Models of discrete choice data are grounded in the theoretical underpinnings of the characteristic theory of value (Lancaster 1966) and the random utility theory (McFadden 1974). An individual chooses an improved cook stove since he/she values the attributes of the product. Here, we assume utility as a latent construct that underlies observed choices reflecting the demand for a good. Respondent *n* is assumed to consider the full set of offered alternatives *i* and choosing the alternative with the highest utility. As implied by the characteristic theory of value, utility of option *i* for individual *n* (U_in_) is assumed to depend on the attributes (*Z*_*i*_) of the good to be valued and the socioeconomic characteristics of individual users (*S*_*n*_). This utility is decomposed into deterministic and stochastic components as:

1 $$ {U}_{in}={V}_{in}+{\varepsilon}_{in} $$

where U_in_ is the latent, unobserved utility of consumer *n* for choice alternative *i*, V_in_ is the deterministic part of the utility that individual *n* has for choice alternative *I*, and ε_in_ is the random portion of the utility that consumer *n* has for choice alternative *i*. Employing the rationality assumption, that is, individuals are utility maximizers, the probability that individual ^*n*^ will choose option ^*i*^ over ^*j*^ is given by:

2 $$ \mathrm{Prob}\left(i|R\right)=\mathrm{Prob}\left({U}_{in}>{U}_{jn}\right)=\mathrm{Prob}\left({V}_{in}+{\varepsilon}_{in}>{V}_{jn}+{\varepsilon}_{jn}\right)\ \mathrm{for}\  j\epsilon R\ \mathrm{and}\ i\ne j $$

where R is the complete choice set available to the individual. This probability is estimable under the assumption that the error terms are independently and identically distributed with extreme-value distribution. This assumption gives rise to the specification of the multinomial logit model (MNL) that determines the probabilities of choosing alternative ^*i*^ over ^*j*^ (Hanley et al. 2001):

3 $$ Prob\left({U}_{in}>{U}_{jn}\right)=\frac{\exp \left(\upmu {\mathrm{V}}_{\mathrm{i}}\right)}{\sum \exp \left(\upmu {\mathrm{V}}_{\mathrm{j}}\right)};\forall \mathrm{j}\ne \mathrm{i} $$

where *V*_*i*_ = *V*(*Z*_*i*_, *S*) is the indirect utility function, *Z*_*i*_ is a vector of the energy-efficient cook stove’s attributes, *S* is a vector of users’ socioeconomic characteristics, and *μ* is a scale parameter inversely related to the standard deviation of the error term. The implication of this is that the estimated βs cannot be directly interpreted for their contribution to utility since they are confounded with the scale parameter. The MNL model must satisfy independence from irrelevant alternatives’ ^(*IIA*)^ properties, which means that the addition or subtraction of any alternatives to the choice available to the respondents will not affect the relative probability of individual *n* choosing any other alternative (Hausman and McFadden 1984). The deterministic utility function from the MNL model can be presented by *V*_*ij*_ which is assumed to have an additive structure and is given by:

4 $$ {V}_{ij}= ASC+\sum {\beta}_k{Z}_k+\sum {\beta}_m{S}_m $$

where ASC (alternative-specific constant) captures systematic variations in choice observations which are associated with alternatives that are not explained either by an attribute’s variations or by respondents observed socioeconomic characteristics (Ben-Akiva and Lerman 1985). It accounts for the effects of any attribute not included in the choice set for utility (Agimas and Mekonnen 2011). *β*_*k*_ is a vector of coefficients corresponding to Z’s attributes from the choice sets, and *β*_*m*_ is a vector of coefficients corresponding to S_m_ socioeconomic characteristics of the respondents. To improve the efficiency of the estimated coefficients and WTP, the study used the bootstrap method (Cameron and Trivedi 2010; Takama et al. 2011). Once the multinomial logit model’s estimations were obtained, the marginal value of each attribute and the corresponding marginal rates of substitution were estimated. Besides, a measure of welfare change (compensating surplus) that conforms to the demand theory can be derived from the proposed changes in the improved cook stove’s attributes. The marginal rate of substitution for each attribute is estimated following Hanley et al. (2001):

5 $$ \mathrm{WTP}={\mathrm{b}}_{\mathrm{y}}^{-1}\ln \left\{\frac{\sum_{\mathrm{i}}\exp \Big({\mathrm{V}}_{\mathrm{i}}^1}{\sum_{\mathrm{i}}\exp \Big({\mathrm{V}}_{\mathrm{i}}^0}\right\} $$

where V_i_^0^ represents the utility of the initial state and V_i_^1^ represents the utility of the alternative state. The parameter b_y_ is the coefficient of price attribute which measures the marginal utility of income. For the linear utility index representation, WTP is simply the ratio of the coefficient of an attribute to the coefficient of payment (price). This ratio is called part-worth/implicit price or marginal willingness to pay for the attribute. It is estimable in Nlogit using the Krinsky and Robb method (Hensher et al. 2005):

6 $$ \mathrm{WTP}=-\frac{\beta_{attribute}}{\beta_{payment}} $$

The assumption of IIA in the MNL model is hard to meet in real choice models. If assumption of IIA is violated (which can be tested using the Hausman and McFadden (1984) procedure), we have to resort to models which relax this assumption. One such model is the random parameter logit (RPL) model also known as the mixed logit model (Train 1998). As opposed to the MNL model, the mixed logit model (MLM) allows for high flexibility by specifying taste coefficients to be randomly distributed across individuals, and it accounts for households’ unobserved heterogeneity in decision-making (Campbell et al. 2011; Hensher and Greene 2003; Kroon et al. 2014). It also allows for interdependence of the choice situation by allowing the AR(1) process. MLM generalizes a standard MNL by allowing its parameters associated with the observed variable to vary with a known population distribution across individuals. It also assumes continuous joint distribution and is specified as:

7 $$ {P}_{iqt}=\frac{\exp \left({\alpha}^{\prime }+{\beta}^{\prime }{X}_{iqt}+{\varphi}^{\prime }{F}_{i\mathrm{q}t}\right)}{\sum_{j=1}^J\exp \left({\alpha}^{\prime }+{\beta}^{\prime }{X}_{jqt}+{\varphi}^{\prime }{F}_{jqt}\right)} $$

where α′ is a vector of fixed or random ASCs associated with i = 1,…, J alternatives and q = 1,…, Q individuals and one of these ASCs should be identified as 0. β′ is a parameter vector that is randomly distributed across individuals. φ′ is a vector of non-random parameters. X_iqt_ is a vector of individual-specific characteristics and alternative-specific attributes at choice situation t and is estimated with random parameters. F_iqt_ is a vector of individual-specific characteristics and alternative-specific attributes at choice situation t and is estimated with fixed parameters. As an alternative to the mixed logit model which assumes continuous joint distribution of the sources of individual preference heterogeneity, we also use the latent class model which assumes discrete distribution of preference heterogeneity.

Following Boxall and Adamowicz (2002) and Greene and Hensher (2003), we use a latent class model for analyzing the behavior of different classes in choosing energy-efficient cook stoves. Here, a consumer shows discrete preference heterogeneity for goods and services. This consumer preference heterogeneity must be accounted for while estimating a consumer’s WTP. The latent class model’s specification stands on the theory that individual behavior depends on observable attributes and on latent heterogeneity that varies with factors that are unobserved by an analyst (Greene and Hensher 2003; Shen 2009). This heterogeneity can be analyzed by the model of discrete parameter variation. The central assumption in this model is that individuals are implicitly sorted into a set of Q classes and the analyst has no prior information about the class to which an individual belongs. Following Greene and Hensher (2003), the latent class model can be extended from the central behavioral model of the multinomial logit model for discrete choices among J_i_ alternatives by individual i observed in T_i_ choice situations:

8 $$ \mathrm{Prob}\left[\mathrm{that}\ \mathrm{individual}\ \mathrm{i}\ \mathrm{chooses}\ \mathrm{j}\ \mathrm{i}\mathrm{n}\ \mathrm{choice}\ \mathrm{situation}\ \mathrm{t}|\mathrm{class}\ \mathrm{q}\right]=\frac{\exp \left({\mathrm{X}}_{\mathrm{it},\mathrm{j}}^{\prime }{\upbeta}_{\mathrm{q}}\right)}{\sum_{\mathrm{j}=1}^{\mathrm{J}}\exp \left({\mathrm{X}}_{\mathrm{it},\mathrm{j}}^{\prime }{\upbeta}_{\mathrm{q}}\right)}=\mathrm{F}\left(\mathrm{i},\mathrm{j},\mathrm{t}|\mathrm{q}\right) $$

Consequently, the probability of a specific choice made by an individual i given the class to which s/he belongs can be specified as:

9 $$ {\mathrm{P}}_{\mathrm{it}\mid \mathrm{q}}\left(\mathrm{j}\right)=\mathrm{Prob}\left({\mathrm{y}}_{\mathrm{it}}=\mathrm{j}|\mathrm{class}=\mathrm{q}\right) $$

In this formulation, for the given class assignment, the contribution of individual i to the likelihood function will be the joint probability of the sequence *y*_*i*_ = [*y*_*i*1_, *y*_*i*2_, …, *y*_*iT*_] given as:

10 $$ {\mathrm{P}}_{\mathrm{i}\mid \mathrm{q}}={\prod}_{\mathrm{t}=1}^{{\mathrm{T}}_{\mathrm{i}}}{\mathrm{P}}_{\mathrm{i}\mathrm{t}\mid \mathrm{q}} $$

The class assignment is not known, but if H_iq_ denotes the prior probability for class q for individual i, it can conveniently be presented as a multinomial logit as:

11 $$ {\mathrm{H}}_{\mathrm{i}\mathrm{q}}=\frac{\exp^{\left({\mathrm{Z}}_{\mathrm{i}}^{\prime }{\uptheta}_{\mathrm{q}}\right)}}{\sum_{\mathrm{q}=1}^{\mathrm{Q}}{\exp}^{\Big({\mathrm{Z}}_{\mathrm{i}}^{\prime }{\uptheta}_{\mathrm{q}}}\Big)},\mathrm{q}=1,2,\dots, \mathrm{Q},{\uptheta}_{\mathrm{Q}}=0 $$

where z_i′_s are a set of observable characteristics which enter the model of class membership. To enable identification of the model, the Qth parameter is normalized to zero. The likelihood of individual i is the expectation (over classes) of class-specific contributions given as:

12 $$ {P}_i={\sum}_{q=1}^Q{H}_{iq}{P}_{1\mid q} $$

Finally, the log likelihood function for the sample can be specified as:

13 $$ lnL={\sum}_{i=1}^N\mathit{\ln}{P}_i={\sum}_{i=1}^N\mathit{\ln}\left[{\sum}_{q=1}^Q{H}_{iq}\left({\prod}_{t=1}^T{P}_{it\mid q}\right)\right] $$

The coefficients are estimated for all classes through maximizing the likelihood function. However, the striking fact here is that the determination of the number of classes for the latent class model is not straightforward. This study examines the optimum class size using the information criteria and comes up with two classes for estimating this model.

Currently, researchers who prefer analysis and discrete choice models have come up with more sophisticated and advanced models which account for both preference heterogeneity and scale heterogeneity. The generalized multinomial logit model (GMNL) is one such model. Following Fiebig et al. (2010), the GMNL model for a sample of *n* respondents with the choice of *J* alternatives in *T* choice situations represents the probability of respondent *i* choosing alternative *j* in choice situation t as:

14 $$ \Pr \left({\mathrm{choice}}_{\mathrm{i}\mathrm{t}}=\mathrm{j}|{\upbeta}_{\mathrm{i}}\right)=\frac{\exp \left({\upbeta}_{\mathrm{i}}^{\prime }{\mathrm{X}}_{\mathrm{i}\mathrm{t}\mathrm{j}}\right)}{\sum_{\mathrm{k}=1}^{\mathrm{J}}\exp \left({\upbeta}_{\mathrm{i}}^{\prime }{\mathrm{X}}_{\mathrm{i}\mathrm{t}\mathrm{j}}\right)},\mathrm{i}=1,\dots, \mathrm{N};\mathrm{t}=1,\dots, \mathrm{J} $$

where *x*_*itj*_ is a vector of observed attributes of alternative *j*, and *β*_*i*_ is a vector of individual-specific parameters defined as:

15 $$ {\upbeta}_{\mathrm{i}}={\upsigma}_{\mathrm{i}}\upbeta +\left\{\upgamma +{\upsigma}_{\mathrm{i}}\left(1-\upgamma \right)\right\}{\upeta}_{\mathrm{i}} $$

Here, the specification of β_i_ is central to GMNL. It depends on a constant vector *β*, a scalar parameter *γ*, a random vector η_i_ distributed multivariate normal, MVN(0, Σ), and σ_i_, the individual-specific scale of the idiosyncratic error. The value of γ ranges from [0, 1], and at extreme values of γ, we get GMNL type I or II. GMNL is able to account for “extreme” consumers with nearly lexicographic preferences. It is able to explain consumers who exhibit very “random” behavior. This paper extends the analysis of the improved cook stove choices to the generalized mixed logit model.

#### Estimating WTP distribution

Estimating WTP for goods and services serves several purposes, and hence, it has been used in different areas. There are several methods for estimating WTP. The first and simplest method is to directly ask the respondents their willingness to pay for the good/service under consideration. However, this method has several problems: respondents may face cognitive difficulties and they may behave strategically while responding to different incentives (Hole and Kolstad 2012). This can be estimated either in the preference (utility space) or in the WTP space. The distribution of WTP in the utility space can also be specified. The utility of household n derives from the use of cook stove j under situation t specified as a function of income of household w_njt_ and other non-income attributes of the stove x_njt_ written as:

16 $$ {U}_{njt}={\alpha}_n{w}_{njt}+\beta {\prime}_n{X}_{njt}+{\varepsilon}_{njt} $$

From Eq. (16), α_n_ and β_n_ indicate individual-specific coefficients for income and the other attributes of the improved cook stove chosen, and ε_njt_ is a random error term. We assume that ε_njt_ is the extreme value distributed with variance given by ${\mu}_n^2\left(\frac{\pi^2}{6}\right)$, where μ_n_ is an individual-specific scale parameter. Train and Weeks (2005) show that dividing Eq. (16) by μ_n_ does not affect the behavior and results in a new error term which is i.i.d. extreme value distributed with variance equal to $\frac{\pi^2}{6}$ and we get a new equation:

(17) ${U}_{njt}=\frac{\alpha_n}{\mu_n}{w}_{njt}+\frac{\beta_n^{\prime }}{\mu_n}{X}_{njt}+{\varepsilon}_{njt}$, which can be written as:

18 $$ {U}_{njt}={\lambda}_n{w}_{njt}+C{\prime}_n{X}_{njt}+{\varepsilon}_{njt} $$

Equation (18) is a specification of WTP in the preference space (Train and Weeks 2005). Moreover, decision-makers are highly influenced by one characteristic of a product during purchase decisions. In the choice experiment, this is known as “attribute non-attendance.” It is an attribute that is not considered by decision-makers or is ignored while making a choice; this has occupied a prominent place in discrete choice models (Hensher et al. 2012). Our study examined the presence of attribute non-attendance in the improved cook stove choice decisions. It used the attribute non-attendance where respondents were asked to state to what extent they had attended to each attribute after the choice exercise was complete.

An analysis of the double-bounded response data from the contingent valuation method (CVM) survey uses interval data. Information which is directly elicited is a dichotomous response taking a value of zero if the individual says no and one if the individual answers yes to a question. Individual I’s response depends on the price (bid) for the product with improvements proposed to be provided. Let us assume that an individual is asked if he is willing to pay t_i_ for a given change in the good proposed to be provided. If the individual’s response is no, then we can say his willingness to pay is between zero and t_i_, that is, 0 ≤ WTP ≤ t_i_ and if he answers yes, his willingness to pay is t_i_ ≤ WTP < ∞. For getting an accurate estimate of WTP, we need a relatively larger sample size which is hardly attainable in such a survey.

Alternatively, the double-bounded methodology proposed by Hanemann et al. (1991) can be used for efficiently estimating WTP. In this case, a respondent is given a follow-up question after he responds yes or no to the first bid question. If the individual answers yes to the first question, he is provided with a higher bid. On the other hand, if the individual answers no to the first bid question, then he is offered a lower bid. For examining the individual’s WTP distribution following Lopez-Feldman’s (2012) explanation, we can denote the first bid price by t^1^ and the second bid price by t^2^. In this regard, each respondent will fall in one of the following categories:

- If an individual answers yes to the first bid question and no to the second bid question, then t^2^ > t^1^. In this case, we can say that t^1^ ≤ WTP ≤ t^2^.
- If an individual answers yes to both the questions, then we have t^2^ ≤ WTP < ∞.
- If an individual answers no to the first question and yes to the second question, then t^1^ > t^2^, and we have t^2^ ≤ WTP ≤ t^1^.
- Finally, if an individual answers no to both the first and second questions, we have 0 < WTP < t^1^.

These four cases can be estimated by the double-bounded or interval data model. To specify the model, let us denote the answer to the first and second response questions by dichotomous variables y_i_^1^ and y_i_^2^. The probability that an individual will answer yes to the first question and no to the second question is given as Pr(y_i_^1^ = 1, y_i_^2^ = 0| z_i_) = Pr(s, n). Specifying this probability distribution works under the assumption that:

WTP_i_(z_i_, u_i_) = Z′_i_β + u_i_ and u_i_~N(0, σ^2^), then the probability distribution of the four choices is:

- Case 1: y_i_^1^ = 1 and y_i_^2^ = 0

19 $$ \Pr \left(\mathrm{yes},\mathrm{no}\right)=\mathrm{pr}\left({\mathrm{t}}^1\le \mathrm{WTP}<{\mathrm{t}}^2\right) $$

$$ =\mathrm{pr}\left({\mathrm{t}}^1\le {\mathrm{Z}}_{\mathrm{i}}^{\prime}\upbeta +{\mathrm{u}}_{\mathrm{i}}<{\mathrm{t}}^2\right) $$

$$ =\mathrm{pr}\Big(\frac{\ {\mathrm{t}}^1-{\mathrm{Z}}_{\mathrm{i}}^{\prime}\upbeta}{\upsigma}\le {\mathrm{u}}_{\mathrm{i}}<\frac{{\mathrm{t}}^2-{\mathrm{Z}}_{\mathrm{i}}^{\prime}\upbeta}{\upsigma} $$ )

$$ =\Phi \left(\frac{\ {\mathrm{t}}^2-{\mathrm{Z}}_{\mathrm{i}}^{\prime}\upbeta}{\upsigma}\right)-\Phi \left(\frac{\ {\mathrm{t}}^1-{\mathrm{Z}}_{\mathrm{i}}^{\prime}\upbeta}{\upsigma}\right) $$

where the last expression follows Pr(a ≤ X < b) = F(b) − F(a). Therefore, using the symmetry of the normal distribution, we have:

20 $$ \Pr \left(\mathrm{yes},\mathrm{no}\right)=\Phi \left({\mathrm{Z}}_{\mathrm{i}}^{\prime}\frac{\upbeta}{\upsigma}-\frac{{\mathrm{t}}^1}{\upsigma}\right)-\Phi \left({\mathrm{Z}}_{\mathrm{i}}^{\prime}\frac{\upbeta}{\upsigma}-\frac{{\mathrm{t}}^2}{\upsigma}\right) $$

- Case 2: y_i_^1^ = 1 and y_i_^2^ = 1

21 $$ \Pr \left(\mathrm{yes},\mathrm{yes}\right)=\Pr \left(\mathrm{WTP}>{\mathrm{t}}^1,\mathrm{WTP}>{\mathrm{t}}^2\right) $$

$$ =\Pr \Big({\mathrm{Z}}_{\mathrm{i}}^{\prime}\upbeta +{\mathrm{u}}_{\mathrm{i}}>{\mathrm{t}}^1,{\mathrm{Z}}_{\mathrm{i}}^{\prime}\upbeta +{\mathrm{u}}_{\mathrm{i}}>{\mathrm{t}}^2 $$

Using Baye’s rule, which says that Pr(A,B) Pr(A|B)*Pr(B), we have:

22 $$ \Pr \left( yes, yes\right)=\mathit{\Pr}\left({Z}_i^{\prime}\beta +{u}_i>{t}^1|{Z}_i^{\prime}\beta +{u}_i>{t}^2\right)\ast \mathit{\Pr}\left({Z}_i^{\prime}\beta +{u}_i>{t}^2\right) $$

Here by definition t^2^ > t^1^ and then $\Pr \left({\mathrm{Z}}_{\mathrm{i}}^{\prime}\upbeta +{\mathrm{u}}_{\mathrm{i}}>{\mathrm{t}}^1|{\mathrm{Z}}_{\mathrm{i}}^{\prime}\upbeta +{\mathrm{u}}_{\mathrm{i}}>{\mathrm{t}}^2\right)=1$ which implies

$$ \Pr \left(\mathrm{yes},\mathrm{yes}\right)=\Pr \left({\mathrm{u}}_{\mathrm{i}}>{\mathrm{t}}^2-{\mathrm{Z}}_{\mathrm{i}}^{\prime}\upbeta \right)=1-\Phi \left(\frac{{\mathrm{t}}^2-{\mathrm{Z}}_{\mathrm{i}}^{\prime}\upbeta}{\upsigma}\right) $$

So, by symmetry we have:

23 $$ \Pr \left(\mathrm{yes},\mathrm{yes}\right)=\Phi \left({\mathrm{Z}}_{\mathrm{i}}^{\prime}\frac{\upbeta}{\upsigma}-\frac{{\mathrm{t}}^2}{\upsigma}\right) $$

- Case 3: y_i_^1^ 0 and y_i_^2^ = 1

24 $$ \Pr \left(\mathrm{no},\mathrm{yes}\right)=\Pr \left({\mathrm{t}}^2\le \mathrm{WTP}<{\mathrm{t}}^1\right) $$

$$ =\Pr \left({\mathrm{t}}^2\le {\mathrm{Z}}_{\mathrm{i}}^{\prime}\upbeta +{\mathrm{u}}_{\mathrm{i}}<{\mathrm{t}}^1\right) $$

$$ =\Pr \left(\frac{{\mathrm{t}}^2-{\mathrm{Z}}_{\mathrm{i}}^{\prime}\upbeta}{\upsigma}\le \frac{{\mathrm{u}}_{\mathrm{i}}}{\upsigma}<\frac{{\mathrm{t}}^1-{\mathrm{Z}}_{\mathrm{i}}^{\prime}\upbeta}{\upsigma}\right) $$

$$ =\Phi \left(\frac{{\mathrm{t}}^1-{\mathrm{Z}}_{\mathrm{i}}^{\prime}\upbeta}{\upsigma}\right)-\Phi \left(\frac{{\mathrm{t}}^2-{\mathrm{Z}}_{\mathrm{i}}^{\prime}\upbeta}{\upsigma}\right) $$

$$ \Pr \left(\mathrm{no},\mathrm{yes}\right)=\Phi \left({\mathrm{Z}}_{\mathrm{i}}^{\prime}\frac{\upbeta}{\upsigma}-\frac{{\mathrm{t}}^2}{\upsigma}\right)-\Phi \left({\mathrm{Z}}_{\mathrm{i}}^{\prime}\frac{\upbeta}{\upsigma}-\frac{{\mathrm{t}}^1}{\upsigma}\right) $$

- Case 4: y_i_^1^ = 0 and y_i_^2^ = 0

(25) Pr(no, no) = Pr(WTP < t^1^, WTP < t^2^)

$$ =\Pr \left({\mathrm{Z}}_{\mathrm{i}}^{\prime}\upbeta +{\mathrm{u}}_{\mathrm{i}}<{\mathrm{t}}^1,{\mathrm{Z}}_{\mathrm{i}}^{\prime}\upbeta +{\mathrm{u}}_{\mathrm{i}}<{\mathrm{t}}^2\right) $$

$$ =\Pr \left({\mathrm{Z}}_{\mathrm{i}}^{\prime}\upbeta +{\mathrm{u}}_{\mathrm{i}}<{\mathrm{t}}^2\right) $$

$$ =\Phi \left(\frac{{\mathrm{t}}^2-{\mathrm{Z}}_{\mathrm{i}}^{\prime}\upbeta}{\upsigma}\right)=1-\Phi \left({\mathrm{Z}}_{\mathrm{i}}^{\prime}\frac{\upbeta}{\upsigma}-\frac{{\mathrm{t}}^2}{\upsigma}\right) $$

For these four models, we have to construct the likelihood function to directly obtain β and σ through the maximum likelihood estimation method. To be maximized and for finding the parameters of the model, the likelihood function can be constructed as:

26 $$ {\sum}_{\mathrm{i}=1}^{\mathrm{N}}\left[\begin{array}{c}{\mathrm{d}}_{\mathrm{i}}^{\mathrm{yes},\mathrm{no}}\ln \left(\Phi \left(\frac{\ {\mathrm{t}}^2-{\mathrm{Z}}_{\mathrm{i}}^{\prime}\upbeta}{\upsigma}\right)-\Phi \left(\frac{\ {\mathrm{t}}^1-{\mathrm{Z}}_{\mathrm{i}}^{\prime}\upbeta}{\upsigma}\right)\right)+{\mathrm{d}}_{\mathrm{i}}^{\mathrm{yes},\mathrm{yes}}\ln \left(\Phi \left(\frac{\ {\mathrm{t}}^1-{\mathrm{Z}}_{\mathrm{i}}^{\prime}\upbeta}{\upsigma}\right)\right)\\ {}.\\ {}+{\mathrm{d}}_{\mathrm{i}}^{\mathrm{no},\mathrm{yes}}\ln \left(\Phi \left(\frac{{\mathrm{t}}^1-{\mathrm{Z}}_{\mathrm{i}}^{\prime}\upbeta}{\upsigma}\right)-\Phi \left(\frac{{\mathrm{t}}^2-{\mathrm{Z}}_{\mathrm{i}}^{\prime}\upbeta}{\upsigma}\right)\right)+{\mathrm{d}}_{\mathrm{i}}^{\mathrm{no},\mathrm{no}}\ln \left(\Phi \left(\frac{{\mathrm{t}}^2-{\mathrm{Z}}_{\mathrm{i}}^{\prime}\upbeta}{\upsigma}\right)\right)\end{array}\right] $$

where d_i_^yes,no^, d_i_^yes,yes^, d_i_^no,yes^, and d_i_^no,no^ are indicator variables that take values of one or zero depending on the relevant case for each individual whereby each individual will only appear once in the likelihood function. Using this likelihood function, estimating the parameters $\left(\hat{\beta\ }\mathrm{and}\hat{\sigma}\right)$ is straightforward.^5^

Finally, this paper tested one method for reducing a hypothetical bias in the contingent valuation survey. Biases abound in contingent valuation studies. People’s intentions and actions deviate in the real world. These can be strategic bias, hypothetical bias, and starting point bias. Several methods are proposed and implemented in contingent valuation literature to reduce these biases. Hypothetical bias is broadly defined as a difference between stated and revealed WTP. It is a systematic divergence between welfare estimates obtained through the stated preference and revealed preference choice instruments. Usually, WTPs stated by the individuals often exceed their real-money WTPs (List and Gallet 2001; Murphy et al. 2005). This paper used cheap-talk to test its impact on reducing the hypothetical bias in the contingent valuation survey. Cheap-talk is communication between players that does not directly affect the pay-offs of the game. In such a game, providing and receiving information is free.

## Results and discussion

### Descriptive statistics

This section presents some descriptive statistics of the responses to some of the questions in the survey. It also briefly presents respondents’ perceptions, awareness, socioeconomic characteristics, and ownership status. It is aimed to give readers some clues about the nature of the data and its distribution. Table 3 gives summary statistics for some of the continuous variables.

**Table 3:** Descriptive statistics of continuous variables (n = 307)

Variables	Mean	Std. dev.
Age of the respondent (in years)	42.82	12.90
Education level of the respondent (in years)	2.96	3.57
Household size (in number)	6.35	2.44
Adult male equivalence household size	5.05	2.05
On-farm employed members	2.10	1.49
Off-farm employed members	0.14	0.37
Land owned in local units (kert)	7.27	6.54
Land cultivated in local units (kert)	9.39	8.23
Land rented in local units (kert)	3.28	6.03
Land rented out in local units (kert)	0.34	1.42
Number of oxen owned	2.01	1.56
Livestock owned (in TLU)	5.25	3.91
Household expenditure per month (in birr)	1541.68	980.79
Household income per month (in birr)	2115.57	1661.49

Source: Authors’ computations using survey data

As can be seen in Table 3, on average, the age of the respondents was 42.82 years with a standard deviation of 12.90 years. Household heads in the study area, on average, achieved grade 3, and family size was about six which is relatively higher than the family size in rural Ethiopia. On average, sample households’ monthly income was about 2116 birr, and monthly expenditure was slightly lower than the income at about 1542 birr. Off-farm employment was not common in the study area. Respondents’ land ownership status shows that, on average, a household possessed about 7.27 kert (less than 2 ha) of land. It seems that landholding in the study area did not meet the land demands of a majority of the farmers as they were, on average, net renters-in. Oxen were predominantly used as a source of power for plowing. On average, a household owned two oxen and about 5.25 livestock in terms of tropical livestock units.

Table 4 shows that close to two-thirds of the respondents did not have access to credit. Regarding gender of household heads, about 67.4% of the households were male-headed. The penetration rate of education in rural parts in developing countries is very low. In case one wants to talk about education in these areas, it is more appropriate to talk about literacy rates. About 60% of the household heads in the study area could read and write.

**Table 4:** Frequency of categorical variables

Variables	Category	Frequency	Percentage
Access to credit	Yes	116	37.79
	No	191	62.21
Respondent’s sex	Male	207	67.43
	Female	100	32.57
Can read and write	Yes	183	59.61
	No	124	40.39
Owns a private tree	Yes	265	86.32
	No	42	13.68
Marital status	Married	274	89.25
	Otherwise	33	10.75

Source: Authors’ computations using survey data

More than half of the respondents had experienced different health problems as a result of their current cook stove (Table 5). Physical burns, respiratory diseases, symptoms of asthma, and irritation in the eyes and nose were some of the problems that they had experienced. The results in Table 5 show that a majority of the respondents (about 95%) had information about the improved cook stove. Their sources of information included health extension workers in the area, agricultural development workers, friends, radio, and local community organizations like Idir and Iqub. Despite this information, the penetration rate of the improved cook stove was very low. Only about 26% of the respondents were using the improved cook stove. The responses to a follow-up question showed that the respondents were conservative in using the product due to a bad experience and also because they had very little information about using the product.

**Table 5:** Frequency and distribution of use, health effects, and information about the cook stove

Variables	Category	Frequency	Percentage
Have you had any health-related injury when cooking on a traditional stove?	Yes No	195 112	63.52 36.48
Have you heard about the improved cook stove?	Yes No	290 17	94.46 5.54
Are you currently using any improved cook stove?	Yes No	79 228	25.73 74.27
Do you use the same type of stove for cooking all meals?	Yes No	184 123	59.93 40.07

Source: Authors’ computations using survey data

The results in Table 6 give a clear picture of respondents’ perceptions about using the improved cook stove. Use of the traditional cook stove has tremendous negative health effects. Indoor air pollution, physical burning, and damage to different body parts due to fire and smoke are common health effects of using the traditional cook stove. In line with this, households strongly believed that use of the improved cook stove will reduce these problems. About 62.5% of the respondents strongly agreed that the use of the improved cook stove will improve the health of household members. A majority of the respondents also agreed that the improved cook stove saved time. They also agreed that use of the improved cook stove should be mandatory in Ethiopia to alleviate the problems of using the traditional (three-stone) cook stove.

**Table 6:** Households’ perceptions about the improved cook stove

Variables	Strongly agree (in % )	Agree (in % )	Undecided (in %)	Disagree (in %)	Strongly disagree (in %)
Use of the improved cook stove improves your family’s health.	62.54	30.62	6.84	0	0
Use of the improved cook stove saves time.	62.540	32.25	5.21	0	0
It is easy to buy the improved cook stove in your area.	54.40	20.85	6.19	13.36	5.21
Fixing the improved cook stove is easy.	39.41	37.46	13.68	8.47	0.98
Use of the improved cook stove should be mandatory in Ethiopia.	61.24	29.97	5.86	2.93	0

Source: Authors’ computation using survey data

Theoretically, as price increases, demand for a product decreases, other things remaining constant. As presented in Table 7, the proportion of respondents who accepted the offered price of the stove (bid) decreased as its price increased. This is in compliance with the theory of valuation which states that as the bid value increases respondents’ willingness to accept the product decreases.

**Table 7:** Responses to distribution and the price of the stove (bid)

Bid in ETB	No	Yes	Total
75	26	79	105
150	50	50	100
250	62	40	102
Total	138	169	307

Source: Authors’ computations using survey data

It is evident from Table 8 that attribute non-attendance was not a serious problem in this choice experiment. A fairly significant number of respondents attended to almost all the attributes while making choice decisions. Fuel wood saving was considered less in the choice exercise. This can be attributed to the fact that rural households are not constrained by the availability of fuel wood.

**Table 8:** Stated attribute non-attendance

	Attended to attribute (percentage of respondents)				
Attributes	Always	Often	Sometimes	Rarely	Never
Emission reduction	36.16	21.17	28.34	12.70	1.63
Fuel wood saving	27.69	18.24	33.88	19.22	0.98
Reducing risk of fire	48.53	21.17	24.76	4.56	0.98
Durability of the stove	50.49	20.85	21.50	6.51	0.65

Source: Authors’ computations using survey data

### Econometric results

A starting point for discrete choice models, the multinomial logit model (MNL) and the mixed logit model (MLM), is running the basic model with only alternative-specific attributes as explanatory variables (Hensher and Greene 2003). Table 9 presents the results of the basic MNL, MLM, the scale heterogeneity multinomial logit model (SMNL), and the generalized mixed logit model (GMNL). Column 2 of Table 9 gives the results of the MNL model. The overall fit of the model as measured by McFadden’s Pseudo R^2^ is good (0.20), but using the log likelihood ratio test and information criterion, it performs poorly compared to the other models. The coefficients of this model are significant at the less than 1% significance level except for fuel wood saving. This indicates that all the selected attributes except fuel wood saving significantly affected the choice of the improved cook stove.

**Table 9:** Results of the various discrete choice models

Variables	Multinomial logit (MNL)		Mixed logit model (MIXL)		Scale heterogeneity (SMNL)		Generalized multinomial logit (GMNL)	
	Coefficient	SE	Coefficient	SE	Coefficient	SE	Coefficient	SE
ASC	0.89***	0.30	3.19***	0.56	1.89**	0.92	3.98***	0.69
Emission reduction	0.24***	0.05	0.28**	0.12	0.22***	0.06	0.44***	0.14
Fuel saving	0.07	0.06	0.07	0.10	0.06	0.07	−0.04	0.12
Risk of fire	0.84***	0.05	1.41***	0.12	0.94***	0.09	1.50***	0.27
Durability of the stove	0.08***	0.01	0.11***	0.01	0.09***	0.01	0.13***	0.02
Price of the stove	−0.002***	0.001	**−**0.003***	0.001	−0.002***	0.001	−0.002***	0.001
SD emissions			1.36***	0.17			1.25***	0.23
SD fuel			0.85***	0.19			0.90***	0.17
SD RISK			0.91***	0.14			1.40***	0.20
SD durability			0.10***	0.01			0.06***	0.01
Tau (τ)					0.46***	0.15	0.42*	0.24
Gamma (γ)					0.00	0.00	0.55	0.42
Sigma (σ_i_)					1.00**	0.46	1.00**	0.42
No. of parameters	6		10		9		13	
Log likelihood	−1112.20		−999.01		−1110.20		−1002.90	
Pseudo R^2^	0.20		0.51		0.45		0.50	
AIC	2236.50		2018.00		2234.40		2029.80	
BIC	2269.60		2073.20		2273.00		2096.00	
HQIC	2248.70		2038.40		2248.70		2054.20	

Note: ***, **, and * show significance at the 1%, 5%, and 10% level of significance

However, the MNL model is estimated under a stringent assumption of IIA. This study tested this assumption by using the Hausman test by excluding one of the alternatives. The chi-square value of 17.96 with a p-value of 0.006 shows that the IIA assumption was violated in the MNL model. As a result, this study estimated the alternative model which relaxes this assumption. The first model is the random parameter logit model, also known as the mixed logit model. The merits of this model not only are relaxing the assumption of IIA, but it also considers preference heterogeneity. The results of the mixed logit model are presented in column 4 of Table 9. The estimates of standard deviations show that there is preference heterogeneity in choosing the improved cook stove.

The results in Table 9 show that for all the models, the coefficient of an alternative-specific constant is positive and significant. The positive and significant alternative-specific constant (ASC) coefficient indicates that respondents had higher utility for policy alternatives (improved cook stove) compared to the status quo which is similar with the findings of Jagger and Jumbe (2016) but contrasts with the findings of Jeuland et al.’s (2015) study which showed that consumers exhibited a stronger baseline preference for the traditional stove. As expected, the coefficient of price was negative and significant indicating that respondents had higher utility for alternatives with lower price levels. The positive and significant coefficients of emission reduction, reducing risks of use, and the durability of the stove show that the respondents derived higher utility from the stove with lower levels of emissions, lower risks of fire, and longer durability of the stove. Our findings are similar to that of Takema et al.’s (2011) and Kooser’s (2014) findings. Stove-specific attributes are important in determining the adoption of the improved cook stove. In our study, the fuel-saving feature of the improved cook stove became insignificant in all the models that we estimated which is different from Kooser’s (2014) findings. This might be due to cultural and multiple benefits of fire wood use in rural parts as besides cooking, households might use fire wood for lighting and heating. Furthermore, easy accessibility to fire wood in rural parts might make this attribute insignificant unlike in urban areas (Bielecki and Wingenbach 2014).

The coefficients of the scale heterogeneity multinomial logit model (SMNL) confirm the existence of scale heterogeneity (significant tau). Furthermore, the results of the generalized multinomial logit model show the existence of both scale and preference heterogeneity. Thus, accounting for these will improve the efficiency of the estimated coefficients.

The mixed logit model assumes continuous preference heterogeneity in estimating random parameters. This, however, may not always be true in the real world where people may exhibit discrete preference heterogeneity. Thus, this paper estimated the latent class model to allow a discrete change in preferences. Optimum class size was selected using the information criterion. Since the third and fourth classifications bring no improvements to the model’s fit, the two-class model was estimated. The results of this model are given in Table 10.

**Table 10:** Results of the latent class model

Attributes/class	Class 1	Class 2
Utility functions		
ASC	−0.076	−3.143
	(0.453)	(2.056)
Emission reduction	0.245***	−0.151
	(0.057)	(0.353)
Fuel wood saving	0.100	−0.983**
	(0.063)	(0.431)
Reducing fire risks	0.863***	0.643**
	(0.055)	(0.316)
Durability of the cook stove	0.085***	0.044
	(0.006)	(0.043)
Price of the cook stove	−0.001***	−0.016***
	(0.0005)	(0.005)
Class membership function:		
Constant	16.669***	
	(5.838)	
Income	0.005***	
	(0.0015)	
Age	−0.325***	
	(0.105)	
Latent class probs.	0.961	0.039
Number of obs.	1842	
Log likelihood	−1074.22	
Pseudo R^2^	0.47	

Note: ***, **, and * show significance at the 1%, 5%, and 10% level of significance

Finally, from the estimated models, the study computed marginal willingness to pay (MWT) for each attribute. This result carries important policy implications as cook stove designers, producers, and policymakers can clearly identify the most important features of the cook stove. The marginal rate of substitution (Table 11) shows the rate at which a consumer trades off one attribute for another.

**Table 11:** Marginal willingness to pay (MWT) for the improved cook stove’s attributes using the MNL model

Model/attributes	Emission reduction	Fuel saving	Risk reduction	Durability
MWT_MNL	117.98***	36.49	472.58***	46.35***
Std. dev.	(42.98)	(35.61)	(127.18)	(12.39)

As we can see in Table 11, the respondents made a trade-off when they took decisions about adopting the improved cook stove. They attached more value to the emission reduction attribute of the improved cook stove followed by reduction in the risks of a fire. Their marginal willingness to pay was lower for the fuel-saving attributes of the proposed cook stove.

This paper also examined the determinants of willingness to pay for the improved cook stove using data generated by the contingent valuation survey. For this, it analyzed the double-bounded contingent valuation data using the interval data model. For comparison purposes, we estimated the probit model using initial bid and the biprobit model using both responses to initial and follow-up bids and the interval data model. The results of the probit model are given in Table 12. As expected, when the bid is increased, the probability of saying yes decreases. The educational level of a household head, household income, livestock ownership, and non-farm income positively affected a household’s willingness to pay for the improved cook stove.

**Table 12:** Determinants of WTP’s results from CVM data

Variables	Coef.	Std. err.
Stove price (bid)	−0.08***	0.002
Age of the respondent (log)	0.04	0.39
Education level	0.06*	0.03
Family size (AME)	−0.16**	0.06
Income (log)	0.69***	0.20
Livestock (TLU)	0.18***	0.05
Off-farm employment	−0.48*	0.28
Non-farm employment	0.15*	0.09
Land owned	−0.03	0.02
Access to credit	0.26	0.21
Own a private tree	0.38	0.28
Cheap-talk	−0.64***	0.21
Constant	−2.95	2.11
Pseudo R^2^	0.29	
Likelihood ratio test, chi^2^ test value	85.20 (p-value 0.000)	

Note: ***, **, and * represent coefficients significant at the 1%, 5%, and 10% level respectively

The price of the stove (bid), family size, and off-farm employment negatively and significantly affected the adoption of the improved cook stove. This is similar to Mamuye et al.’s (2018) and Takema et al.’s (2011) findings. As expected, provision of cheap-talk reduced willingness to pay. This means that households who received the questionnaire with cheap-talk as an option stated lower willingness to pay compared to those without the cheap-talk option. Thus, cheap-talk can help reduce hypothetical bias in contingent valuations.

After estimating the determinants of willingness to pay for adopting the improved cook stove, this study also estimated mean willingness to pay from the different models estimated. The results of mean willingness to pay are presented in Table 13.

**Table 13:** Results of mean willingness to pay using different models

Variable/model	Probit	Probit with covariates	Doubleleb	Doubleleb model with covariate
Mean willingness to pay	353.87***	208.56***	276.56***	152.66***
Standard error	47.72	44.75	10.45	27.72

As we can see in Table 13, mean willingness to pay for the improved cook stove ranged between 152.66 birr and 353.87 birr. The actual market price of the improved cook stove in Ethiopia is higher than the estimated mean willingness to pay. Lack of affordability of the improved cook stove might discourage its adoption and deprive households of its multiple benefits—health benefits due to reduction in indoor air pollution, cost reduction because of energy efficiency, and a reduction of environmental problems such as deforestation and CO_2_ emissions. It will be possible to harness the multiple benefits of the improved cook stove if the improved cook stove can be made available at a lower cost. This suggests that a dissemination intervention for the improved cook stove requires price support (subsidization policy) for it to be effective.

## Conclusion and policy recommendations

This study used two stated preference survey techniques for analyzing households’ willingness to pay and their preferences for the improved cook stove in Ethiopia. It used different discrete choice models for estimating the determinants of the use of the improved cook stove from data generated using the choice experiment survey. Households exhibited scale and preference heterogeneity when making choice decisions. Our study used the scale heterogeneous multinomial logit model (SMLM) and the generalized mixed logit model (GMLM) to account for this heterogeneity. Our results show the existence of scale and preference heterogeneity as seen in a significant tau coefficient. None of the existing studies on the topic have used these models and their results might be biased.

Our study also tested attribute non-attendance which is a behavioral response which could affect the estimated marginal rate of substitution among the different attributes. Using the stated attribute non-attendance, the study verified that households attended to all attributes when taking choice decisions. Fuel wood saving was less attended to among all the attributes. Our study also used cheap-talk to reduce the hypothetical bias from the contingent valuation exercise. Households who had received the questionnaire with cheap-talk as an option stated a lower mean willingness to pay compared to those without the cheap-talk option. Therefore, use of cheap-talk in contingent valuation will significantly reduce hypothetical bias.

Our results show that cook stove–related attributes and the respondents’ socioeconomic characteristics were important in determining preferences for adopting the improved cook stove. Emission reductions, lower risks of use, and durability of the stove positively affected the probability of households adopting the improved cook stove. Our results are similar to Mamuye et al.’s (2018) and Takema et al.’s (2011) findings. However, Bielecki’s and Wingenbach’s (2014) study stresses the importance of considering determinants other than socioeconomic and stove-related ones of the improved cook stove. Rural households value stoves beyond their cooking needs such as for lighting, heating, and as a gathering point for the family. Thus, stove designers need to consider these aspects too as they affect the adoption and dissemination of improved cook stoves. Among the attributes, the estimated marginal rates of substitution showed the trade-off that households made while deciding about purchasing the improved cook stove. Thus, cook stove designers, producers, and pricing policies should take this trade-off into account for effective dissemination of the improved stove. The estimated results of the scale heterogeneous multinomial logit model clearly show the existence of scale heterogeneity and the generalized multinomial logit model’s coefficients show that there are both scale and preference heterogeneities. Thus, failure to account for these factors will underestimate the coefficients of product-related and socioeconomic characteristics in estimating willingness to pay for the improved cook stove.

Our results also show that the sampled respondents were aware of the side effects of traditional energy sources and their health, environmental, and economic consequences. They were interested in adopting and using the improved cook stove but were frustrated by low-quality products and related inconveniences of using the limited existing products. Thus, designers of the improved cook stove should make it simple and convenient for use fitting the realities of rural housing and stove use. Reliability of the stove should also be ensured to enhance its uptake.

The estimated mean willingness to pay ranged from about 150 Birr to 350 Birr. This shows that the respondents’ willingness to pay was below the supply price of the improved cook stove in the area. Affordability of the improved cook stove might discourage its adoption and deprive households of its multiple benefits. Making the improved cook stove affordable for low-income rural households requires different price stabilizations. Capitalizing on the multiple benefits of the improved cook stove price subsidization can be obtained from sources such as carbon financing of the clean development mechanism.

## Data Availability

The decoded data and codes are available on request.

## Footnotes

1 http://www.worldenergyoutlook.org/resources/energydevelopment/energyaccessdatabase/ (accessed on 30 April 2020).

2 https://energypedia.info/wiki/Ethiopia_Energy_Situation (accessed on 30 April 2020).

3 Kebele is the smallest administrative unit, equivalent to a neighborhood with a minimum of 500 households.

4 For saving space, the survey is not included here. However, it is available with the lead author on request.

5 The Doubleleb Stata command is used for estimating this interval data model after adding the ado developed by Lopez-Feldman (2012) in Stata Version 12.
